# Computational exploration of hydrogen storage potential in Mg_2_LiXH_6_ (X = Ti, V) hydrides *via* DFT and AIMD simulations

**DOI:** 10.1039/d6ra01881e

**Published:** 2026-05-19

**Authors:** Malik Muhammad Asif Iqbal, Muhammad Kaleem, Amna Nasir, Asif Nawaz Khan, Muhammad Abaidullah

**Affiliations:** a Department of Chemistry, University of Okara Pakistan mmasif101@gmail.com; b Material Research Laboratory (MRL), International Islamic University H-10 Islamabad 44000 Pakistan mkaleemphy@gmail.com; c Materials Modeling and Simulation Lab, Department of Physics, University of Science & Technology Bannu 28100 Khyber Pakhtunkhwa Pakistan; d Department of Physics, University of Okara Pakistan

## Abstract

Perovskite-based materials offer considerable potential for efficient, stable, and environmentally sustainable hydrogen storage technologies. In this study, an inclusive density functional theory (DFT) investigation was conducted to evaluate the structural, mechanical, electronic, optical, and thermodynamic features of Mg_2_LiXH_6_ (X = Ti, V) double perovskite hydrides. Both compounds adopt a stable cubic *Fm*-3*m* symmetry supported by favorable tolerance factors (0.87 for Ti and 0.92 for V) and negative formation energies (−1.15 and −1.27 eV per atom), confirming their thermodynamic stability. Both compounds exhibit thermal stability at 600 K without significant structural distortion, as confirmed by *ab initio* molecular dynamics (AIMD) simulations. Dynamic stability was evaluated using phonon dispersion calculations, which showed no imaginary frequencies, confirming that the system remains stable at 0 K. Mechanical analysis confirms elastic and Born stability, with Poisson's ratios of 0.31 (Mg_2_LiTiH_6_) and 0.24 (Mg_2_LiVH_6_) and *B*/*G* ratios of 2.35 and 1.61, indicating ductile behavior for Mg_2_LiTiH_6_ and a slightly brittle nature for Mg_2_LiVH_6_. Electronic structure calculations reveal metallic behavior while optical analyses indicate potential for optoelectronic applications. Both materials, Mg_2_LiTiH_6_ and Mg_2_LiVH_6_, exhibit promising hydrogen storage characteristics with gravimetric capacities of 5.28 and 5.14 wt% and estimated desorption temperatures of 425.55 and 467.23 K, respectively. These results showed that Mg_2_LiXH_6_ (X = Ti, V) are promising candidates for next-generation hydrogen storage and energy conversion systems.

## Abstract 2

DFT, phonon, and AIMD investigations reveal that Mg_2_LiXH_6_ (X = Ti, V) double perovskite hydrides possess excellent structural stability, favorable dehydrogenation temperatures, and achieve >5 wt% H_2_ capacity for solid-state hydrogen storage.

## Graphical abstract

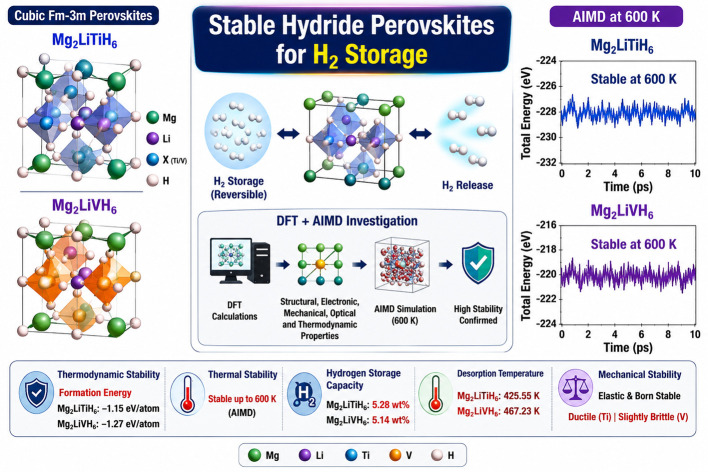

## Introduction

The escalating global energy demand, combined with the rapid depletion of fossil fuel reserves, has intensified the urgency for advanced energy storage systems and sustainable energy sources.^1–3^ Energy scarcity and environmental deterioration are two of the most significant problems facing modern civilization, and they seriously jeopardize the objective of green and sustainable growth. In this context, hydrogen has emerged as a promising clean energy source due to its high energy density and environmental friendliness.^4–6^ Since it is anticipated to be essential to future low-carbon energy systems, it has attracted a lot of study attention. Effective hydrogen storage and transportation are still necessary for the practical deployment of hydrogen technology.^7^ Compressed gas, cryo-compressed liquid, and solid-state storage are examples of current storage techniques. Although the first two approaches are technologically advanced, they require a significant energy input to maintain cryogenic or high-pressure conditions and are vulnerable to boil-off losses from exposure to ambient heat.^8^ Solid-state hydrogen storage, on the other hand, has attracted considerable attention in recent decades due to its inherent safety and advantageous storage properties. However, creating new materials that provide effective and reversible uptake and release of hydrogen continues to be a crucial scientific task.^9^

Metal hydrides, intermetallic alloys^10^ and a broad spectrum of advanced functional materials such as graphene, metal–organic frameworks (MOFs), MXenes,^11–14^ and liquid organic hydrogen carriers are among the most extensively investigated candidates for solid-state hydrogen storage.^6^ Metal hydrides provide high volumetric energy densities and improve operating safety due to their capacity to chemically bond hydrogen at comparatively low pressures. Hydride-based perovskites have garnered a lot of interest because of their distinct physicochemical characteristics and structural stability. It has been demonstrated that adding metal vacancies and transition metal dopants modifies their thermodynamic stability and lowers the hydrogen desorption temperatures, enhancing overall storage performance.^15,16^ Metal hydrides are generally classified into binary hydrides such as LiH_2_, MgH_2_, TiH_2,_ and CaH_2_ (ref. 17) and complex multicomponent systems whose hydrogen storage behavior can be precisely tuned through compositional and structural control.

Perovskite-like hydride systems have demonstrated favorable kinetics and thermodynamics for solid-state hydrogen applications, and perovskite materials have lately surfaced as viable candidates for hydrogen storage beyond their established roles in photovoltaics and optoelectronics. For instance, Tao *et al.* employed high-energy ball milling to synthesize NaMgH_3_ and its potassium-substituted analogue Na_0.9_K_0.1_MgH_3_ (*P*3*m*-type), demonstrating that K-doping enhances the degrading performance of NaMgH_3_.^18^ Similarly, Martínez-Coronado *et al.* used a high-pressure synthesis route to incorporate Li^+^ ions successfully, lowering the hydrogen desorption temperature.^19^ The structural and functional features of double perovskite (DP) hydrides have not yet been thoroughly investigated because to the lack of theoretical and experimental research on the subject. A-site cations inhabit the interstitial spaces in the corner-sharing BX_6_ octahedral framework used by these materials. Such compounds generally have the formula A_2_BXH_6,_ where A is a monovalent or divalent cation, B and X are usually transition metals, and H is an anionic species. Double perovskite hydrides are interesting prospects for uses including energy conversion and hydrogen storage because of their adaptable structure, which provides a rich platform for regulating physicochemical features.^20^

Recent research has brought attention to the structural stability, storage capacity, and optoelectronic behavior of double perovskite hydrides, highlighting their potential for energy harvesting and hydrogen storage. Building on this foundation, a detailed density functional theory (DFT) investigation of the A_2_LiCuH_6_ (A = Be, Mg, Ca, Sr) hydrides with a particular focus on their thermodynamic stability, mechanical robustness, optical performance, and suitability for hydrogen storage.^21^ Using first-principles calculations, Arharbi *et al.* investigated the impact of hydrogen doping and magnesium vacancies on enhancing the thermodynamic and hydrogen storage properties of Li_2_BeMgH_6_ to investigate how such atomic-scale modifications affect the stability and storage efficiency of related hydride systems.^16^ Moreover, Almahmoud *et al.* have reported on the mechanical, electronic, and hydrogen storage properties of Y_2_CoH_6_-type perovskite hydrides, noting their structural integrity and metallic conductivity. By methodically examining the hydrogen storage capability of comparable compounds, their research expands on these discoveries and shows that Mg_2_CoH_6_ in particular reaches a noteworthy gravimetric capacity of 5.32wt% making it a good contender for future solid-state hydrogen storage applications.^22^ Furthermore, recent studies have expanded the exploration of complex hydrides by incorporating diverse combinations of B-site cations. For instance, Ca–Cd-substituted systems, such as X_2_CaCdH_6_ (X = Rb, Cs), have been the focus of research for their structural and hydrogen-storage characteristics.^23^ Moreover, transition-metal-based compounds of the type LiTM_3_LiH_8_ (TM = Sc, Ti, V) have been the focus of study to understand the role of transition metals in tuning the electronic and thermodynamic properties of hydrides.^24^ Considerable attention has been directed towards other related double perovskite and mixed hydride systems. These include A_2_BH_6_ (A = K, Rb; B = Ge, Sn),^25^ K_2_LiX_6_ (X = Al, Ga, In),^26^ and mixed anion/cation compounds, such as KNaMg_2_F_6−*x*_H_*x*_ and KNaAe_2_H_6_ (Ae = Be, Mg, Ca).^27^ Collectively, these studies underscore the mounting interest in compositional tuning as a viable strategy for enhancing structural stability and hydrogen-storage performance in complex hydride materials.

Lithium and magnesium-based hydride perovskites have received a lot of attention because of their potential for hydrogen storage and their attractive functional characteristics. In this study, the structural, mechanical, AIMD, electrical, optical, thermodynamic, and hydrogen storage properties of Mg_2_LiXH_6_ (X= Ti, V) are all thoroughly examined. This B-site chemical configuration remains largely unexplored in double perovskite hydrides and represents a key factor in tuning structural stability and hydrogen-bonding interactions. These materials are strong contenders for next-generation hydrogen storage systems because they demonstrate thermodynamic, mechanical, and kinetic stability. The present findings indicate that these materials exhibit favorable properties for hydrogen storage and offer valuable insights for the development of efficient and sustainable energy storage technologies.

## Computational methodology

The double perovskite hydrides Mg_2_LiXH_6_ (X= Ti, V) were systematically explored using first-principles calculations within the framework of Density Functional Theory (DFT) as implemented in the CASTEP.^28^ The generalized gradient approximation (GGA) with the Perdew–Burke–Ernzerhof (PBE) functional was used for structural optimizations. Vanderbilt-type ultrasoft pseudopotentials were used to explain electron-ion interactions in order to solve the Kohn–Sham equations using the plane-wave pseudopotential approach.^29^ Geometry optimization was conducted using the Broyden–Fletcher–Goldfarb–Shanno (BFGS) minimization algorithm^30^ which effectively ensures convergence of electronic wavefunctions and charge densities in periodic systems. For better self-consistent field (SCF) convergence, the Pulay density mixing approach was utilized.^31^ The Brillouin zone was sampled using a Monkhorst–Pack *k*-point mesh of 6 × 6 × 6, and a kinetic energy cutoff of 600 eV was selected for the plane-wave basis.^32^ All calculations were subject to stringent convergence criteria with an energy change per atom threshold of 2 × 10^−5^ eV and a maximum Hellmann–Feynman force of 0.05 eV Å^−1^ and a maximum ionic displacement of 0.002 Å. Mechanical stability was assessed through the computation of elastic constants and associated mechanical parameters under this optimized condition.

For thermal stability assessment, *ab initio* molecular dynamics was performed by choosing 40 atoms conventional supercell using the pw.x component of Quantum ESPRESSO, integrating trajectories with the velocity-Verlet algorithm in the canonical NVT conditions controlled by a Berendsen thermostat coupling at 600 K. A time step of 0.96756 fs was applied for 12 500 steps, giving a total simulation time of about 12.1 ps. For phonon calculations a 2 × 1 × 1 supercell with 20 atoms was employed using phonopy package integrated with Quantum ESPRESSO. The self-consistent field (SCF) calculations were performed using ultrasoft pseudopotentials and the PBEsol exchange–correlation functional. Plane-wave cutoff energies of 67 Ry for AIMD and 85 Ry for phonon calculations were employed, while the charge-density cutoff was fixed at 560 Ry. The convergence thresholds for SCF iterations were set to 1.0 × 10^−8^ Ry for AIMD and 1.0 × 10^−9^ Ry for phonon calculations. Moreover, the total energy and force convergence criteria were 1.0 × 10^−5^ Ry and 1.0 × 10^−4^ Ry per Bohr, respectively.^32^

## Results and discussion

### Structural properties

**Fig. 1:**
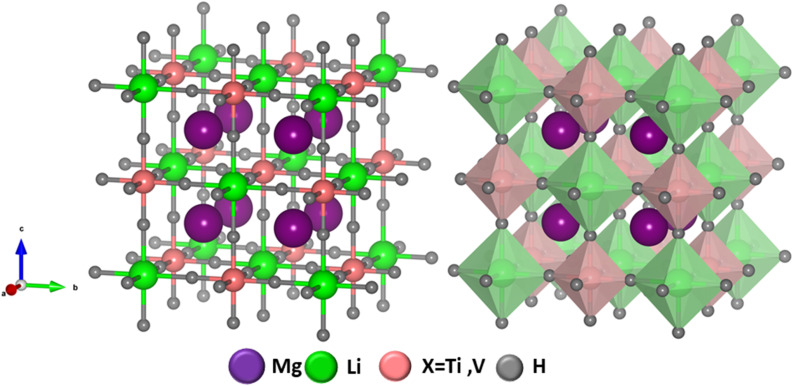
Optimized crystal structure of Mg_2_LiXH_6_ (X = Ti, V) hydrides.

Geometric stability and crystallographic characteristics of Mg_2_LiXH_6_ (X = Ti, V) double perovskite hydrides were examined using DFT. All compounds adopt a cubic lattice structure with the *Fm*-3*m* space group (no. 225) characteristic of double perovskite hydrides. This structure features a central X-site cation encircled by LiH_6_ and AlH_6_ octahedra that share corners.^33^ The primitive unit cell of these structures consists of 10 atoms while the conventional unit cell includes 40 atoms where the atomic positions in the Mg_2_LiXH_6_ (X = Ti, V) compounds are shown in Fig. 1. Specifically the Mg is located at 8c 
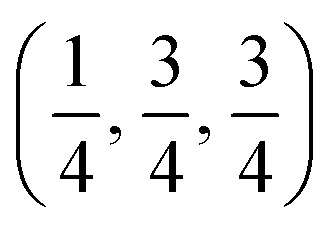
 positions, Li is found at the center 4b 
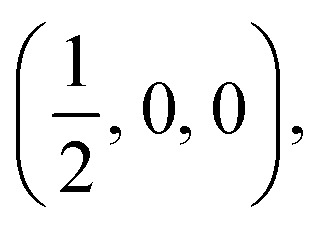
 X = Ti, V is situated at the 4b (0,0,0) and H atoms are placed at 24e 
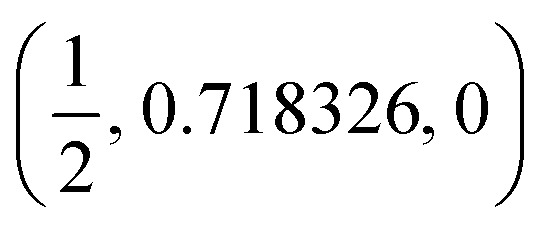
 in accordance with the space group.^21^ According to Table 1, the calculated lattice constants for Mg_2_LiTiH_6_ and Mg_2_LiVH_6_ are 7.17 Å, and 6.98 Å, respectively, indicating an increasing trend with the atomic radius of the alkaline-earth metal.

**Table 1:** Calculated structural parameters of Mg_2_LiXH_6_ (X = Ti, V) hydrides

Compound	Lattice constant *a* = *b* = *c* (Å)	Volume (Å)^3^	*τ*_G_	*µ*	Δ*H*_f_ (eV per atom)	Δ*H*_f_ (kJ per mol per H_2_)	*E*_coh_ (eV per atom)
Mg_2_LiTiH_6_	7.17	368.97	0.87	0.54	−1.15	−55.62	+1.15
Mg_2_LiVH_6_	6.98	340.21	0.92	0.46	−1.27	−61.07	+1.27

The structural stability of the investigated hydride compounds was assessed by calculating the octahedral factor (µ) and Goldschmidt's tolerance factor (*τ*_G_) using the eqn (1) and (2).^34,35^1
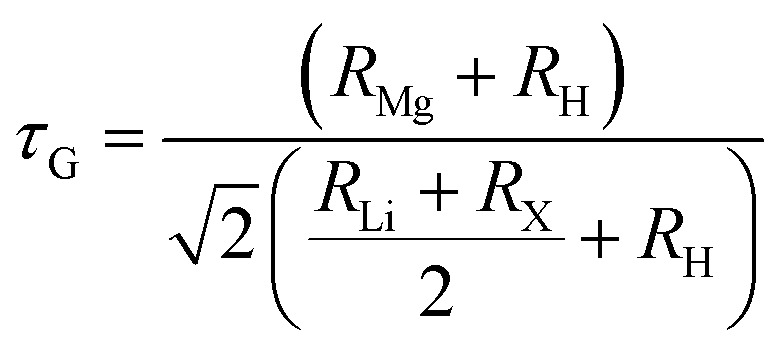
2
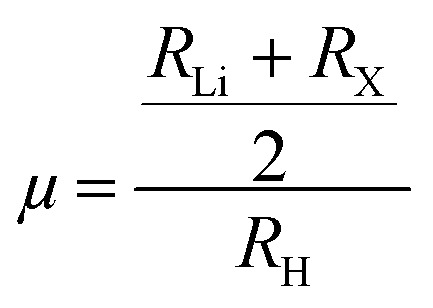
Here, *R*_Mg_, *R*_Li_, *R*_X_ and *R*_H_ represents ionic radii of Mg, Li (X = Ti, V) and H atoms. Stable perovskites typically exhibit a tolerance factor (*τ*_G_) values in the range of 0.71–1.0.^36^ The octahedral factor (µ) a key indicator of structural stability typically lies within the recommended range of 0.42 to 0.75 for stable perovskite configurations.^37^ According to the *τ*_G_ values presented in Table 1 for the investigated compounds, both compounds are confirmed to be structurally stable as the values lie in the optimal range. Also, B and B′ sites are the same for all compounds, so the octahedral factors are the same and lie in the optimal range, which again confirms the structural stability. Moreover, formation energy (Δ*H*_f_) of a compound is one of the key factors influencing its suitability for hydrogen storage. Δ*H*_f_ values have been calculated by using the following eqn (3).^38^3
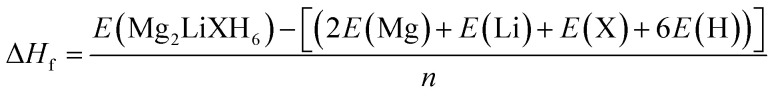


In eqn (3)*E*(Mg), *E*(Li), *E*(X) and *E*(H) denote the total energies of the isolated atoms including magnesium; lithium; X = Ti, V; and hydrogen respectively while *E*(Mg_2_LiXH_6_) corresponds to the total energy of the compound, and *n* represents the total number of elements. The calculated formation energies (Δ*H*_f_) summarized in Table 1 are negative for all three hydrides, confirming their thermodynamic stability and indicating exothermic formation. Among these molecules, Mg_2_LiVH_6_ exhibits the most favorable stability with a formation energy of −1.27 eV, suggesting a strong driving force for synthesis.

To evaluate the relative phase stabilities of Mg_2_LiXH_6_ (X = Ti, V) compounds the cohesive energy (*E*_coh_) was calculated. By measuring the energy required to disintegrate a solid into its constituent isolated atoms this metric represents the strength of interatomic bonding. The calculated (*E*_coh_) values presented in Table 1 were obtained using eqn (4).^39^4
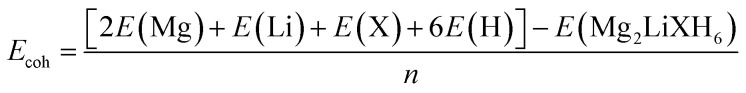


The atomic combination is more stable when the cohesive energy value is larger. According to Table 1, Mg_2_LiAlH_6_ exhibits the highest cohesive energy of +1.27 eV, further supporting the accuracy of the calculated formation energies.

### *Ab initio* molecular dynamics (AIMD) calculations

**Fig. 2:**
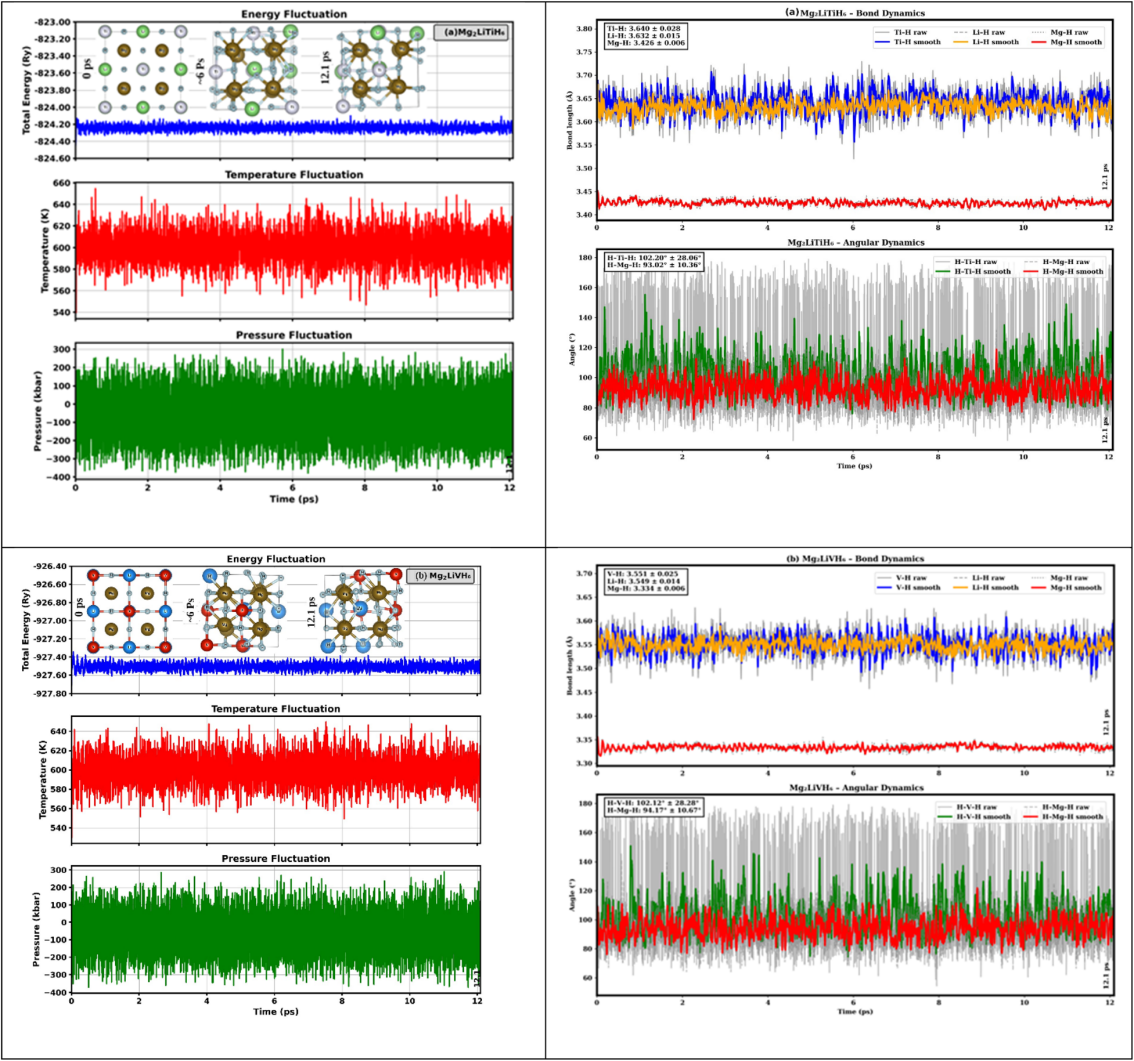
AIMD total-energy traces, bond length and angle dynamics *versus* time for (a) Mg_2_LiTiH_6_ hydrides and (b) Mg_2_LiVH_6_ hydrides.

*Ab initio* molecular dynamics (AIMD) simulations were carried out at 600 K in order to confirm the thermal and dynamical stability of the suggested hydride complexes beyond static total energy calculations.^40^ By integrating first-principles electronic structure simulations with classical atomic motion, AIMD offers a rigorous framework for assessing finite-temperature stability. This allows for real-time monitoring of the development of energy, temperature, and pressure under thermal perturbations. The AIMD trajectories for Mg_2_LiTiH_6_ and Mg_2_LiVH_6_ are displayed in Fig. 2(a and b), respectively.

For Mg_2_LiTiH_6_, the total energy remains well converged throughout the 12.1 ps simulation, fluctuating within a narrow range around −927.50 Ry without any noticeable drift or sudden discontinuities. The temperature oscillates around the target value of 600 K (∼560–640 K), indicating proper thermal equilibration, while the pressure shows small and bounded fluctuations with no systematic divergence. A similar trend is observed for Mg_2_LiVH_6_, where the total energy stabilizes around −824.25 Ry with only minor thermal variations. The temperature and pressure profiles of both systems confirm the absence of mechanical instability or structural degradation during the simulation.^41^

To further confirm structural stability at the microscopic level, a time-dependent analysis of bond lengths and bond angles was performed. The evolution of key bond lengths, including V–H (3.551 ± 0.025 Å), Li–H (3.549 ± 0.014 Å), and Mg–H (3.334 ± 0.006 Å), shows only small fluctuations around their equilibrium values, with no signs of systematic drift, progressive elongation, or bond breaking during the simulation. This behavior confirms that the lattice framework remains stable under thermal excitation. Similarly, the angular distributions remain stable, with H–V–H (102.12° ± 28.28°) and H–Mg–H (94.17° ± 10.67°) fluctuating around their average values without any abrupt distortions. Although some thermal broadening is expected at elevated temperature, the limited range of these fluctuations shows that the local coordination geometry remains preserved.

The combined analysis of total energy, temperature, pressure, bond lengths, and bond angles provides strong evidence that both Mg_2_LiTiH_6_ and Mg_2_LiVH_6_ retain their structural integrity under finite-temperature conditions. The absence of lattice collapse, bond dissociation, or significant angular distortion highlights their robust thermodynamic stability and supports their potential suitability for practical applications, particularly in hydrogen storage and energy-related technologies.^42^

### Phonon dispersion analysis

**Fig. 3:**
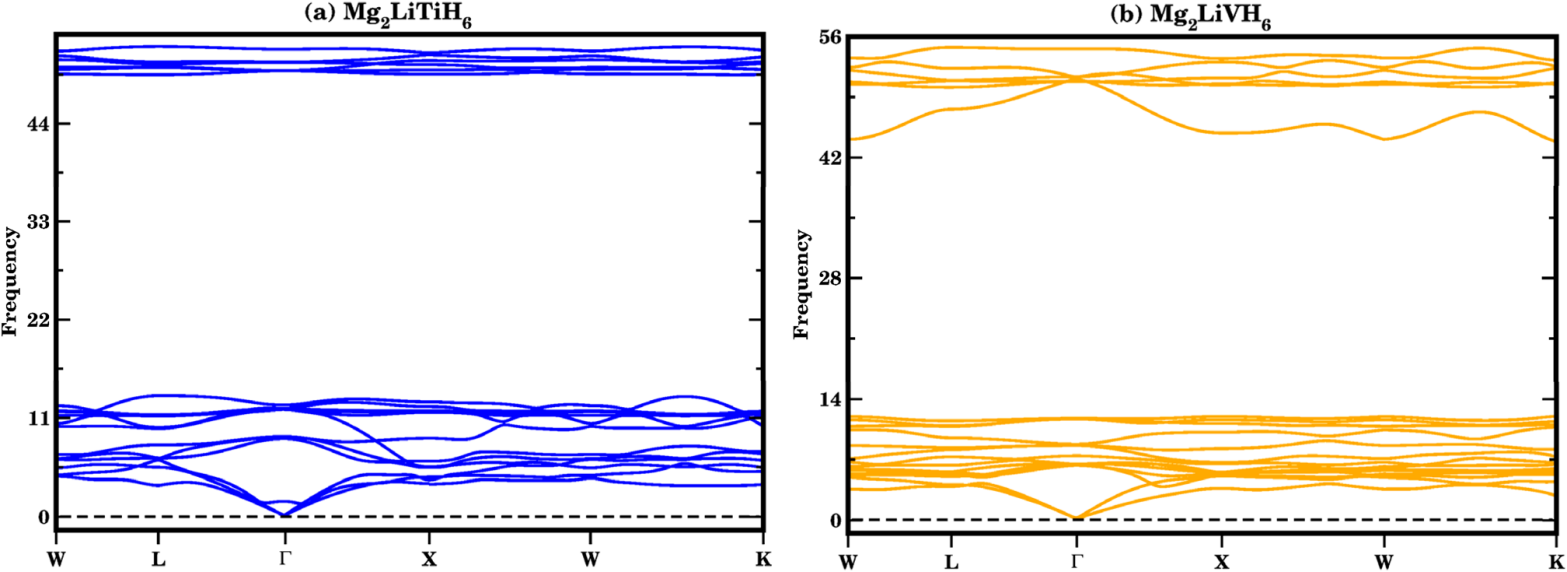
(a and b) Dynamics stability for (a) Mg_2_LiTiH_6_ and (b) Mg_2_LiVH_6_ hydrides.

The dynamic stability of Mg_2_LiTiH_6_ and Mg_2_LiVH_6_ was further examined through phonon dispersion analysis, as shown in Fig. 3(a and b). The phonon spectra of both compounds show no imaginary (negative) frequencies across the entire Brillouin zone, confirming their dynamic stability at 0 K. The absence of soft modes indicates that both crystal structures remain stable against small atomic perturbations and do not undergo spontaneous structural distortions.^43,44^

For Mg_2_LiTiH_6_, the phonon branches are well defined, with low-frequency acoustic modes smoothly transitioning into higher-frequency optical modes without any discontinuities. Similarly, Mg_2_LiVH_6_ shows a stable phonon profile, where all vibrational modes remain positive, further confirming its structural robustness. The relatively high optical phonon frequencies can be attributed to the presence of light hydrogen atoms, which make a significant contribution to lattice vibrations.^20,45^

The phonon dispersion results confirm that both Mg_2_LiTiH_6_ and Mg_2_LiVH_6_ are dynamically stable and satisfy the fundamental stability criterion. When combined with the finite-temperature stability demonstrated by AIMD simulations, these findings provide strong evidence of both harmonic (0 K) and anharmonic (finite-temperature) stability, further supporting the reliability of these materials for practical applications.

### Hydrogen storage characteristics

One of the biggest obstacles to the world's shift to hydrogen-based electricity is still hydrogen storage. For practical applications to proceed, solid-state materials with high gravimetric and volumetric hydrogen densities, advantageous thermodynamic properties, and safe operating temperatures must be developed.^46^ Because of their exceptional capacity and reversibility, complex metal hydrides based on lightweight elements like magnesium, lithium, and transition metals have emerged as particularly intriguing options.^47^ A study of the hydrogen storage potential of perovskite-type hydrides based on Mg_2_LiXH_6_ (X = Ti, V) has been conducted using first-principles calculations in this study, with a focus on their gravimetric and volumetric capacities as well as their behavior regarding hydrogen desorption.

To provide a clearer assessment of performance, a comparative analysis of the advantages and limitations of the investigated hydrides is presented based on their gravimetric capacity, volumetric density, and desorption characteristics. An essential metric for assessing the weight efficiency of hydrogen storage is the gravimetric hydrogen storage capacity (*C*_ωt%_). *C*_ωt%_ measures the amount of hydrogen contained in a material when compared to its total mass. As a percentage, it is calculated by dividing the mass of hydrogen stored by the total mass of the hydrogen storage material using eqn (5).^48^5
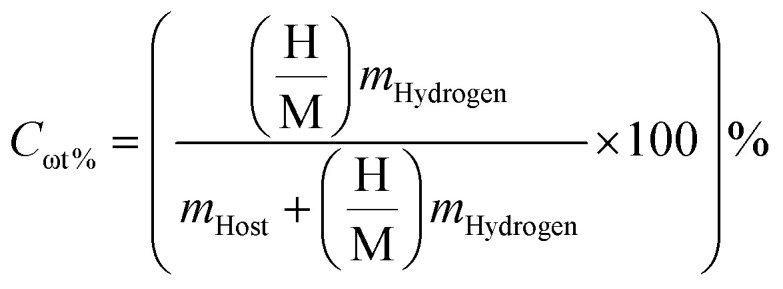
Here *m*_Host_ and *m*_Hydrogen_ represent the molar masses of the host alloy and hydrogen, respectively, and 
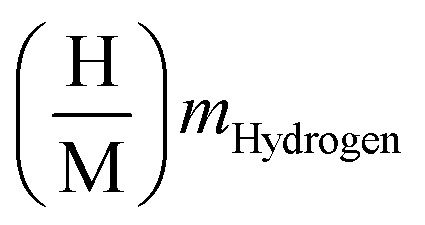
 signifies the ratio of hydrogen atoms to host-material atoms. A higher *C*_ωt%_ value indicates greater hydrogen storage capacity per unit mass, making the material more favorable for practical applications, particularly for on-board hydrogen storage in fuel cell technologies. US Department of Energy (DOE) 2025 targets of a minimum ∼5.5 wt% is recommended for practical on-board applications.^49^ The following are our calculated capacities: Mg_2_LiTiH_6_ (5.28 wt%) and Mg_2_LiVH_6_ (5.14 wt%) (Table 2). In comparison with previously reported double perovskite hydrides, similar trends have been observed. For example, Ayyaz *et al.* reported gravimetric hydrogen capacities of ∼4.5–6.3 wt% for A_2_LiCuH_6_ (A = Be, Mg, Ca, Sr),^21^ while Hakami *et al.* demonstrated higher capacities (∼7.5 wt%) in A_2_FeH_6_ systems due to the presence of lighter elements.^50^ In contrast, systems such as X_2_CaCdH_6_ (X = Rb, Cs) studied by Shahzad *et al.* exhibit lower gravimetric capacities (∼1.39–1.69 wt%)^51^ but improved thermodynamic stability. These comparisons indicate that the hydrogen storage performance of double perovskite hydrides is strongly influenced by the atomic mass and composition of the constituent elements. Although both systems approach the DOE target, Mg_2_LiTiH_6_ is closer to the threshold, indicating greater weight efficiency. This suggests that Mg_2_LiTiH_6_ offers an advantage in terms of gravimetric performance, whereas Mg_2_LiVH_6_ shows slightly lower weight efficiency. Compared with the literature, the present Mg_2_LiXH_6_ systems offer competitive gravimetric capacities while maintaining favorable thermodynamic properties, indicating a balanced hydrogen storage performance. Based on these characteristics, these hydrides may prove to be viable materials for storing hydrogen in a solid state.

**Table 2:** The volumetric and desorption temperatures and gravimetric ratios of Mg_2_LiXH_6_ (X= Ti, V) hydrides

Compound	*T*_des_ (K)	*ρ*_vol_ (g per H_2_ per L)	*C*_ωt%_
Mg_2_LiTiH_6_	425.55	27.219	5.28%
Mg_2_LiVH_6_	467.23	29.519	5.14%

Besides gravimetric efficiency, volumetric capacity (g per H_2_ per L) also plays a key role in the compact design of storage units. There is a direct relationship between the volumetric hydrogen density and the tank design and footprint of the system. The volumetric hydrogen capacities of the studied hydrides are theoretically estimated using the corresponding mathematical eqn (6).^52^6
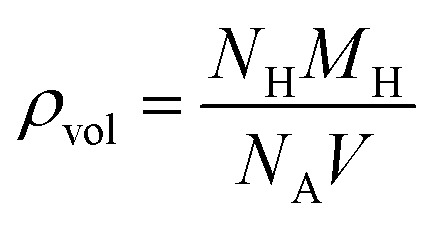


In this relation *N*_H_ represents the number of hydrogen atoms per formula unit, *M*_H_ is the molar mass of hydrogen, *V* denotes the volume of the unit cell and *N*_A_ is Avogadro's number. As a result, the calculated values of Mg_2_LiTiH_6_ → 27.219 g per H_2_ per L and Mg_2_LiVH_6_ → 29.519 g per H_2_ per L (Table 2). Notably, Mg_2_LiVH_6_ outperforms Mg_2_LiTiH_6_ in terms of volumetric performance, making it a more attractive alternative in applications where space constraints are particularly significant such as automotive and aerospace systems. This indicates that Mg_2_LiVH_6_ possesses a clear advantage in volumetric hydrogen storage, whereas Mg_2_LiTiH_6_ shows comparatively lower density. The denser packing of V-based systems may be attributed to a subtle difference in lattice compression or a change in molar volume after hydrogenation.

Desorption temperatures of hydride systems determine whether hydrogen can be reversibly released under ambient or near-ambient conditions. Ideally, a hydrogen storage material should exhibit hydrogen desorption in the temperature range of 300–500 K, making it suitable for both mobile and stationary applications. Using the Gibbs relation *T*_des_ can be calculated using eqn (7).^52^

7 Δ*G* = Δ*H* − *T*_des_·Δ*S*

where Δ*G* represents Gibbs free energy, Δ*H* represents formation enthalpy and Δ*S* represents the entropy change in hydrogen. For solid materials, when metal hydrides decompose at normal pressures and temperatures, only changes in entropy caused by hydrogen evolution are considered. For hydrogen, entropy changes by approximately −130 J mol^−1^ K^−1^ at standard pressures and temperatures. Accordingly *T*_des_ can be calculated using eqn (8).^53^8
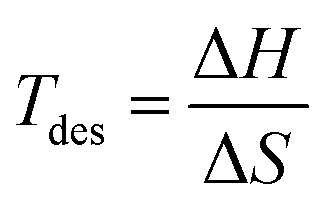


Findings show that the desorption temperature of Mg_2_LiTiH_6_ is 425.55 K, and Mg_2_LiVH_6_ shows a slightly higher value of 467.23 K (Table 2). A technical feasibility window exists within which both values reside, suggesting that hydrogen can be released without the use of extreme thermal input. In Mg_2_LiVH_6,_ the higher desorption temperature may result from enhanced hydrogen lattice binding induced by the more electronegative vanadium dopant, suggesting a stronger hydrogen bonding within the host lattice. This indicates that Mg_2_LiTiH_6_ has an advantage in terms of lower desorption temperature and easier hydrogen release, whereas Mg_2_LiVH_6_ requires comparatively higher thermal input. However, there is a design trade-off between the performances of these two hydrides: as Mg_2_LiTiH_6_ offers much better gravimetric performance as well as lower desorption energy, it is more suitable for use in mobile devices. The Mg_2_LiVH_6_ may find applications in stationary storage systems due to its high volumetric capacity and thermal stability. Overall, Mg_2_LiTiH_6_ is advantageous for weight-sensitive applications, while Mg_2_LiVH_6_ is more favorable for volume-constrained systems, highlighting a clear trade-off between gravimetric efficiency and volumetric density. Having such dual properties makes the Mg_2_LiXH_6_ family a compositionally tunable platform with specific dopants being selected according to application requirements. The results open the door for a more thorough investigation of Mg–Li-based hydride frameworks for clean energy applications and emphasize the significance of transition metal tuning (Ti *vs.* V) in enhancing hydrogen storage qualities.

For Mg_2_LiVH_6_ and Mg_2_LiTiH_6_, the decomposition process may reasonably be expected to follow a stepwise dehydrogenation pathway analogous to that reported for related complex and perovskite hydrides. In the initial stage, partial hydrogen release may occur through disruption of the more weakly bound hydride environments, leading to a partially dehydrogenated intermediate: Mg_2_LiMH_6_ → Mg_2_LiMH_5_ + ½H_2_ (M = V, Ti). With increasing temperature, further destabilization of the hydride framework and progressive collapse of the MH_6_ octahedra may promote decomposition into simpler binary hydrides, which can be described schematically as: Mg_2_LiMH_6_ → 2MgH_2_ + LiH + MH_2_ + ½H_2_. At higher temperatures, these intermediate hydrides may further decompose into their elemental constituents with additional hydrogen evolution (MgH_2_ → Mg + H_2_, LiH → Li + ½H_2_, MH_2_ → M + H_2_), yielding the overall reaction: Mg_2_LiMH_6_ → 2Mg + Li + M + 3H_2_. These stepwise pathways provide a more realistic thermodynamic framework for discussing the stability of these compounds. Importantly, we emphasize that negative formation energies referenced to isolated atoms indicate favorable compound formation relative to free atoms but do not imply thermodynamic stability with respect to competing phases, nor do they guarantee experimental synthesizability. Accordingly, the formation energies reported in this work are interpreted as comparative indicators of bonding and relative stability trends, while a rigorous assessment of synthesizability would require explicit evaluation of decomposition energetics against competing hydrides.

### Mechanical resilience and elastic moduli

Elastic properties play a central role in evaluating the mechanical strength and stability of materials under applied stress.^54^ For the Mg_2_LiXH_6_ (X = Ti, V) hydrides, the second-order elastic constants (*C*_11_, *C*_12_ and *C*_44_) were computed and are listed in Table 3. These constants offer insight into the material resistance to linear, volumetric, and shear deformation. The calculated values confirm that both compositions satisfy the Born stability criteria for cubic crystals, namely: *C*_11_ > 0, *C*_44_ > 0 and *C*_11_ + 2*C*_12_ > 0 (ref. 55) indicating that Mg_2_LiTiH_6_ and Mg_2_LiVH_6_ are mechanically stable.

**Table 3:** The computed elastic constants (*C*_*ij*_) and Born stability criterion Cauchy pressure (*C*_P_) for Mg_2_LiXH_6_ (X= Ti, V) hydrides

Compounds	Elastic constant (*C*_*ij*_)			Born stability criteria				Stability
—	*C*_11_	*C*_12_	*C*_44_	*C*_P_	*C*_11_ > 0	*C*_44_ > 0	*C*_11_ + 2*C*_12_ > 0	—
Mg_2_LiTiH_6_	102.69	34.91	19.64	15.27	102.69	19.64	172.51	Stable
Mg_2_LiVH_6_	122.81	29.99	33.02	−3.03	122.81	33.02	182.79	Stable

In Mg_2_LiTiH_6,_ the elastic constants were obtained as *C*_11_ = 102.69 GPa, *C*_12_ = 34.91 GPa and *C*_44_ = 19.64 GPa. In contrast, Mg_2_LiVH_6_ showed comparatively higher values with *C*_11_ = 122.81 GPa, *C*_12_ = 29.99 GPa and *C*_44_ = 33.02 GPa. The mechanical stability is further confirmed by the positive values of *C*_11_ + 2*C*_12_ which were found to be 172.51 GPa for Mg_2_LiTiH_6_ and 182.79 GPa for Mg_2_LiVH_6_. Additionally, the Cauchy pressure is positive for Mg_2_LiTiH_6_ (15.27 GPa), indicating ductile behavior, whereas for Mg_2_LiVH_6_ it is negative (−3.03 GPa) suggesting brittle characteristics.^56^

Further mechanical parameters were evaluated from the elastic constants and are presented in Table 4. These include the bulk modulus (*B*), shear modulus (*G*), and Young's modulus (*E*), which are essential for understanding the response of materials to external forces calculated employing the following eqn (9)–(14).^57–59^9
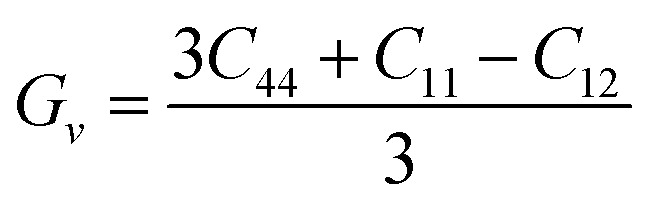
10
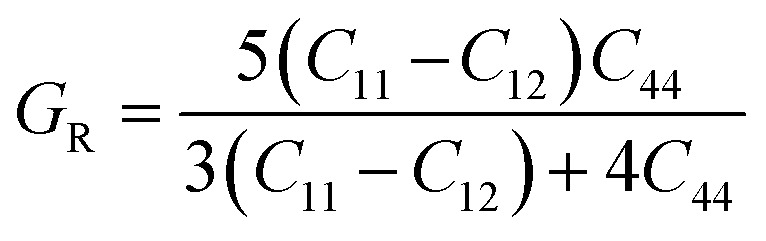
11
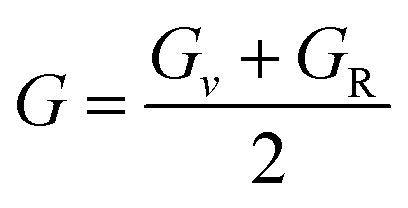
12
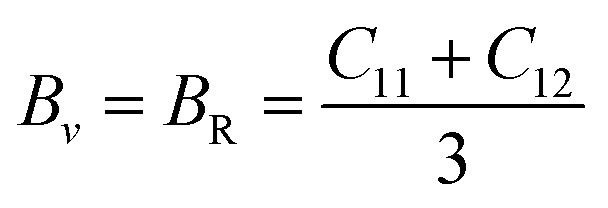
13
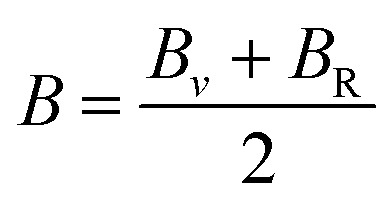
14
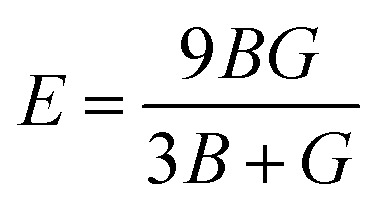


**Table 4:** Elastic constants calculated for Mg_2_LiXH_6_ (X = Ti, V) hydrides

Compounds	*B* (GPa)	*G* (GPa)	*E* (GPa)	*B*/*G*	*ν*	*C*′	*A* ^U^
Mg_2_LiTiH_6_	57.50	24.48	64.31	2.35	0.31	33.89	0.37
Mg_2_LiVH_6_	60.93	37.85	94.08	1.61	0.24	46.41	0.14

Mg_2_LiVH_6_ exhibited higher values for all three moduli: *B* = 60.93 GPa, *G* = 37.85 GPa, and *E* = 94.08 GPa, suggesting higher stiffness and better resistance to deformation compared to Mg_2_LiTiH_6,_ which showed *B* = 57.50 GPa, *G* = 24.48 GPa, and *E* = 64.31 GPa (Table 4). To assess the ductility or brittleness of the materials, Pugh's ratio (*B*/*G*) and Poisson's ratio (*ν*) were evaluated. According to established criteria, materials with *B*/*G* > 1.75 and *ν* > 0.26 exhibit a ductile nature.^60^ Mg_2_LiTiH_6_ satisfies these conditions with *B*/*G* = 2.35 and *ν* = 0.31, while Mg_2_LiVH_6_ shows values of *B*/*G* = 1.61 and *ν* = 0.24, indicating a transition towards brittleness.

The anisotropy of the materials was evaluated through the Zener anisotropy factor (*C*′ = (*C*_11_ − *C*_12_)/2) and the universal anisotropy index 
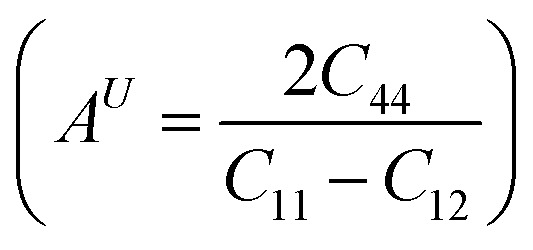
 both of which offer insights into the directional dependence of elastic behaviour. The calculated values of *C*′ are 33.89 GPa and 46.41 GPa for Mg_2_LiTiH_6_ and Mg_2_LiVH_6,_ respectively. The universal anisotropy index *A*ᵁ was found to be 0.37 for Mg_2_LiTiH_6_ and 0.14 for Mg_2_LiVH_6_. These values indicate that both materials exhibit elastic anisotropy, with Mg_2_LiTiH_6_ being slightly more anisotropic.

Because of its stronger and more rigid crystal structure, which enables it to survive the high pressures usually involved in hydrogen absorption, our data show that Mg_2_LiVH_6_ seems to have a larger hydrogen storage capacity. But it also becomes more brittle due to this increased rigidity, raising questions about how long it would last under repeated hydrogen cycling. However, Mg_2_LiTiH_6_ exhibits superior ductility and resilience to cracking or structural collapse despite holding a little less hydrogen. These characteristics increase its long-term stability, particularly in systems where materials are frequently exposed to hydrogen absorption and release. Overall, Mg_2_LiVH_6_ may be better suited for high-capacity storage, but Mg_2_LiTiH_6_ offers greater mechanical reliability, which is equally essential for practical hydrogen storage applications.

According to Table 5, elastic characteristics are also used to estimate important thermodynamic parameters such as sound velocities, Debye temperature (*θ*_D_) and melting temperature (*T*_m_). The degree of lattice vibrations in crystalline materials and the strength of interatomic bonding are both revealed by the Debye temperature. Stronger interatomic forces and better heat conductivity are indicated by a larger *θ*_D_ value. The average elastic velocity (*v*_m_) which is determined from the longitudinal (*v*_l_) and transverse velocities (*v*_t_) has a direct impact on the Debye temperature.^61^ The methodology for these calculations follows standard formulations reported in the literature, with the detailed equations presented in eqn (15)–(18).^62,63^15
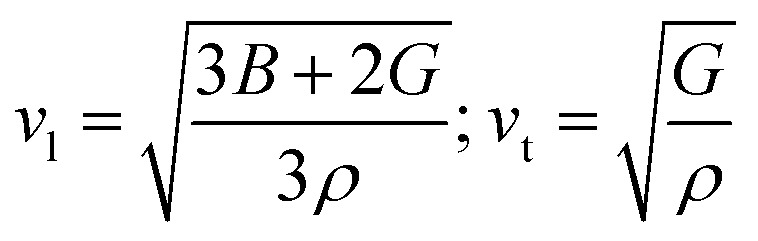
16
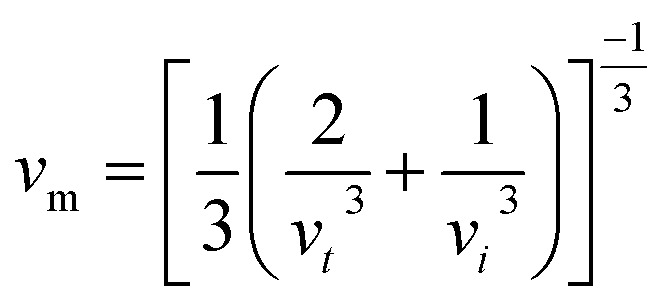
17
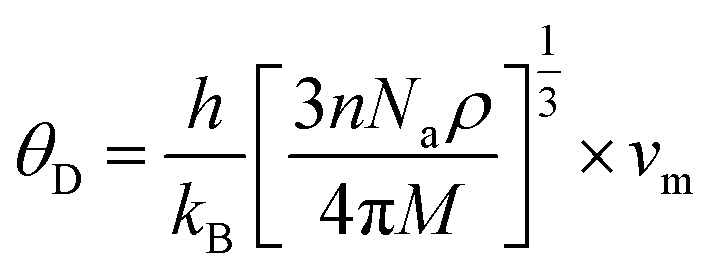

18 *T*_m_ = [553 + 5.911(*C*_11_)] ± 300

**Table 5:** Thermal parameters calculated for X_2_LiAlH_6_ (X = Be/Mg/Ca) perovskite hydrides

Compounds	*v*_l_ (ms^−1^)	*v*_t_ (ms^−1^)	*v*_m_ (ms^−1^)	*T*_m_ (K)	*θ*_D_ (K)
Mg_2_LiTiH_6_	6403.99	3687.82	4095.10	1160.0	248.07
Mg_2_LiVH_6_	6405.48	4245.45	4644.57	1278.93	316.28

The thermodynamic parameters derived from elastic properties provide deeper insight into the lattice dynamics and bonding strength of Mg_2_LiXH_6_ (X = Ti, V). The average sound velocity (*v*_m_) is directly related to the Debye temperature (*θ*_D_) and is determined by the elastic moduli and density of the material. In the present study, Mg_2_LiVH_6_ exhibits higher transverse and average sound velocities than Mg_2_LiTiH_6_, resulting in a higher Debye temperature (316.28 K compared to 248.07 K). This indicates that lattice vibrations in Mg_2_LiVH_6_ occur at higher characteristic frequencies, reflecting a stiffer lattice and stronger interatomic interactions.

Similarly, the estimated melting temperature follows the same trend, with Mg_2_LiVH_6_ (1278.93 K) showing a higher value than Mg_2_LiTiH_6_ (1160.0 K). This behavior can be attributed to its greater elastic stiffness, particularly the higher shear modulus (*G*) and elastic constant *C*_11_, which indicate stronger resistance to shear and longitudinal deformation. These mechanical properties are directly associated with stronger bonding interactions within the crystal lattice.

The substitution of Ti with V enhances the overall rigidity of the lattice, as evidenced by the increased sound velocities and elastic moduli, which consequently raise both *θ*_D_ and *T*_m_. This suggests that Mg_2_LiVH_6_ possesses a more rigid and thermally stable structure. In contrast, the relatively lower Debye temperature and melting point of Mg_2_LiTiH_6_ indicate weaker lattice dynamics, which may facilitate hydrogen diffusion and desorption due to reduced vibrational constraints.

The higher Debye temperature and melting temperature of Mg_2_LiVH_6_ can therefore be consistently attributed to its larger elastic moduli, particularly *C*_11_ and *G*, along with its higher sound velocities, all of which reflect enhanced lattice rigidity. Since the speed of sound is directly proportional to elastic stiffness and inversely proportional to density, the higher values observed for Mg_2_LiVH_6_ indicate stronger resistance to lattice deformation. This increased rigidity leads to higher characteristic vibrational frequencies (*θ*_D_) and requires greater thermal energy to disrupt the lattice (*T*_m_), establishing a physically consistent relationship between mechanical strength and thermal stability. Table 5 suggests a trade-off between thermal robustness and lattice softness within the Mg-based double perovskite hydrides. While Mg_2_LiVH_6_ is thermodynamically more robust and mechanically rigid, Mg_2_LiTiH_6_ may offer advantages for hydrogen release applications where lower lattice stiffness and enhanced vibrational flexibility are desirable. Importantly, mechanical stability is closely related to hydrogen storage performance, as hydride materials undergo repeated lattice expansion and contraction during hydrogen absorption and desorption processes. Materials possessing adequate elastic stiffness and structural integrity are better able to tolerate these volumetric changes without structural degradation. Therefore, the elastic stability of Mg_2_LiTiH_6_ and Mg_2_LiVH_6_ suggests that these hydrides can maintain structural durability during hydrogen cycling, which is an essential requirement for practical hydrogen storage applications.

### Electronic properties

The bonding properties, charge transport behavior, and hydrogen adsorption–desorption kinetics of hydrogen storage materials are significantly influenced by their electronic structure.^64^ First-principles calculations within the GGA-PBE framework were used to carefully evaluate the band structure and density of states of Mg_2_LiTiH_6_ and Mg_2_LiVH_6_ in order to clarify the electronic nature of the researched double perovskite hydrides. While the total and partial density of states provide light on the orbital contributions in charge of bonding and conductivity the computed band structures shed light on the existence or absence of an electronic bandgap.

**Fig. 4:**
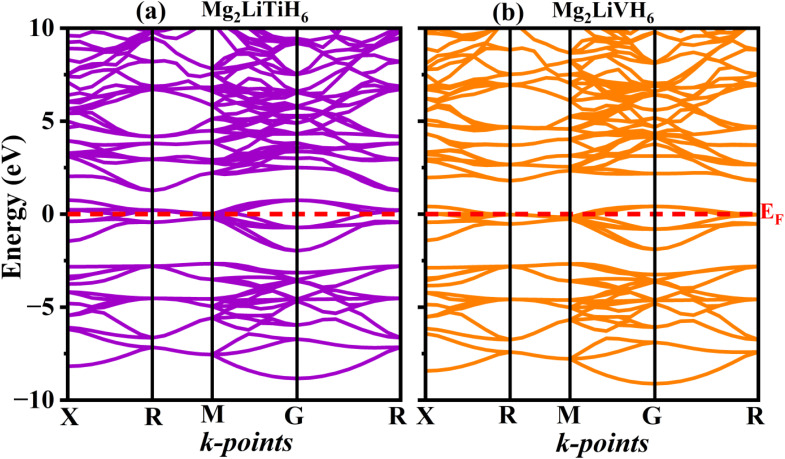
Computed band structures of (a) Mg_2_LiTiH_6_ and (b) Mg_2_LiVH_6_ hydrides.

With the Fermi energy (*E*_F_) aligned at 0 eV, the electronic band dispersions of Mg_2_LiTiH_6_ and Mg_2_LiVH_6_ along the high-symmetry directions *X*–*R*–*M*–*Γ*–*R* are shown in Fig. 4(a and b). Multiple energy bands cross the Fermi level in both compounds, demonstrating a metallic electrical character and suggesting the lack of a bandgap. Delocalized electronic states that promote electronic conductivity are suggested by the constant overlap between valence and conduction bands across the Brillouin zone. Because it improves charge transfer during hydrogen adsorption and desorption processes and encourages reversible hydrogen absorption, such metallic behavior is especially beneficial for hydrogen storage applications.^65^

**Fig. 5:**
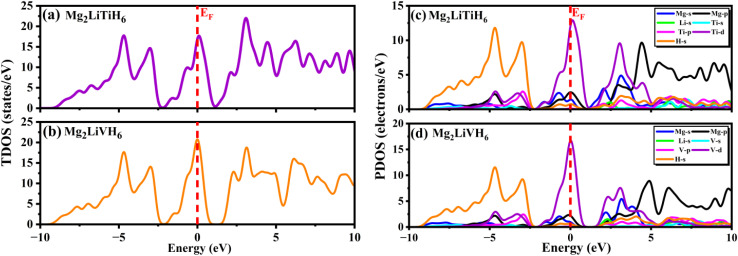
(a–d) TDOS and PDOS of Mg_2_LiXH_6_ (X = Ti, V) hydrides.

Further insight into the electronic stability and bonding characteristics is obtained from the total density of states (TDOS) shown in Fig. 5(a) for Mg_2_LiTiH_6_ and Fig. 5(b) for Mg_2_LiVH_6_. In both cases, the TDOS exhibits a finite density of states at the Fermi level consistent with the metallic nature inferred from the band structures. Crucially, the TDOS near EF has a comparatively broader distribution rather than an overly abrupt peak, indicating electronic stability rather than instability-driven metallicity. This characteristic suggests that intrinsic orbital hybridization, not structural instability, is the cause of metallic behavior.

To identify the orbital origins of the electronic states, the partial density of states (PDOS) was examined and is illustrated in Fig. 5(c) for Mg_2_LiTiH_6_ and Fig. 5(d) for Mg_2_LiVH_6_. Strong hybridization between H-1s states and transition-metal d orbitals (Ti-3d or V-3d) dominates the valence band region in both compounds, which extends from around −6 eV to the Fermi level. Mg-p and Li-s states also contribute. Strong metal–hydrogen bonding, which is necessary for structural stability and hydrogen retention, is reflected in this hybridization. The Ti-3d states in Mg_2_LiTiH_6_ and the V-3d states in Mg_2_LiVH_6_ are the main contributors around the Fermi level suggesting that the transition-metal d orbitals are crucial in controlling metallic conductivity. The electronic states in the conduction band area above *E*_F_ are mostly made up of Mg-p, Li-s and transition-metal d orbitals with small contributions from hydrogen s states, indicating the possibility of weak antibonding interactions that could promote hydrogen mobility.

Overall, the combined band structure TDOS and PDOS analyses demonstrate that Mg_2_LiTiH_6_ and Mg_2_LiVH_6_ are intrinsically metallic hydrides characterized by strong metal–hydrogen bonding and delocalized electronic states near the Fermi level. The dominance of transition-metal d orbitals around *E*_F_ coupled with significant H-1s hybridization in the valence band, highlights a favorable electronic environment for reversible hydrogen storage. These electronic characteristics together with the previously established thermodynamic and dynamical stability reinforce the potential of both compounds as promising candidates for hydrogen-based energy applications.

### Optical features

Mg_2_LiTiH_6_ and Mg_2_LiVH_6_ have been studied primarily for their capacity to store hydrogen. However, understanding their optical behavior is crucial for evaluating their interactions with electromagnetic radiation and thermal management.^66^ As evidenced by the electronic band structure of both compounds, both compounds exhibit metallic properties. As a result, their optical responses are dominated by free electron transitions (intrabands), which are typical of conductive hydrides and metallic alloys.

A complex dielectric function (*ε*(*ω*) = *ε*_1_(*ω*) + *iε*_2_(*ω*)) determines a material's optical response which is strongly related to the electronic band structure.^20^ The real part indicates the degree of polarization or the material's response to an external electric field and the imaginary part is representative of the absorption of the incident photons. Furthermore other optical key parameters such as the absorption coefficient 
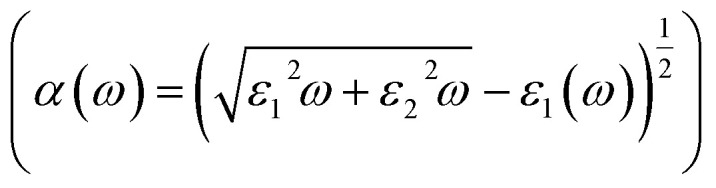
, optical conductivity 
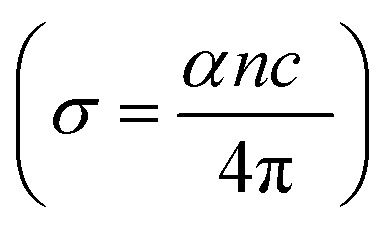
 and reflectivity 
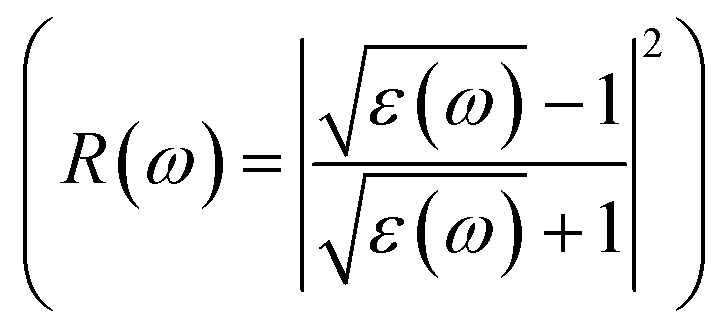
 and all are derivable from the complex dielectric function *ε*(*ω*).^67^

**Fig. 6:**
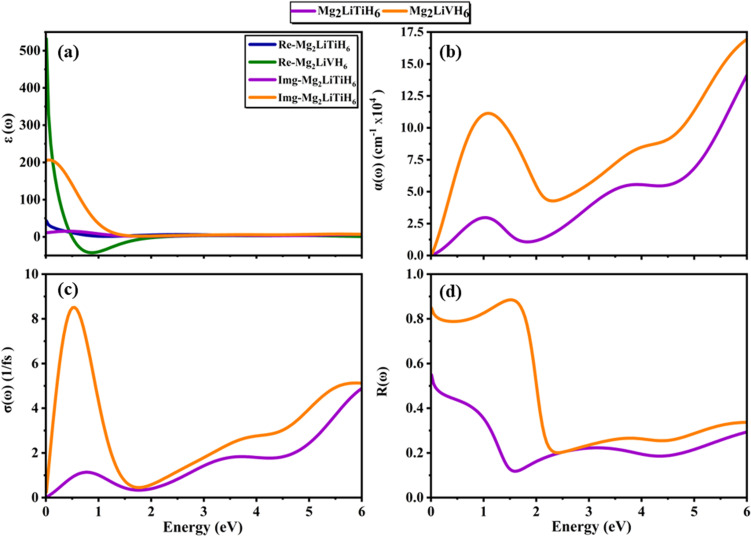
(a) Dielectric constant, (b) absorption coefficient, (c) conductivity and (d) reflectivity of Mg_2_LiXH_6_ (X = Ti, V) hydrides.

Fig. 6(a) illustrates the *ε*_1_(*ω*) exhibiting distinct metallic screening behavior. Mg_2_LiVH_6_ (∼531) is a compound with a very high static dielectric constant as compared to Mg_2_LiTiH_6_ (∼43) which sharply decreases with increasing photon energy. For Mg_2_LiVH_6,_ negative values of *ε*_1_(*ω*) are observed at energies in the range of 0.5 eV to 1.6 eV which reflects plasma-like behavior and suggests a high density of free carriers consistent with Drude-type metals.^68^Fig. 6(a) illustrates the *ε*_2_(*ω*) which quantifies energy absorption due to intraband and interband transitions.^69^ Mg_2_LiVH_6_ shows a prominent peak (∼220) near 0.4 eV and Mg_2_LiTiH_6_ peak at ∼70, indicating strong photon–electron coupling at low photon energies. Metals usually exhibit strong absorption at low photon energies as a result of free carrier excitation and electronic damping.^70^ Both compounds exhibit significant absorption in the 0.3–1.5 eV range peaking near 1.5 × 10^5^ cm^−1^ for Mg_2_LiVH_6,_ as shown in Fig. 6(b).^71^

The optical conductivity can be described as the electrical response to incident photons, which is frequency dependent and related to both electronic transport and light absorption. Fig. 6(c) shows that both compounds exhibit nonzero optical conductivity even at 0 eV, indicating that they are metallic compounds. The much higher conductivity of Mg_2_LiVH_6_ (∼9 1/fs at low energy) is another indication of the larger free carrier and higher optical activation of V doped systems. Beyond 2 eV Drude-like behavior continues to dominate the overall profile for both compounds, while the trend towards higher energy indicates the beginning of interband transitions.

The reflection analysis in Fig. 6(d) provides insight into electromagnetic shielding, thermal emissivity, and surface response of metallic hydrides. Mg_2_LiVH_6_ exhibits high reflectivity (∼0.9) in the infrared region (below 0.5 eV) while Mg_2_LiTiH_6_ is more moderate (∼0.4). An abrupt decrease near 2.0 eV indicates the presence of a plasma resonance edge, after which the material begins to be penetrated by light.^72^

## Conclusion

Density functional theory studies demonstrate that Mg_2_LiXH_6_ (X = Ti, V) double perovskites are suitable for next-generation clean energy systems due to their metallic conductivity, structural stability, and hydrogen storage capabilities. In comparison to conventional Mg-based hydrides such as MgH_2_, which are known for their high thermodynamic stability and correspondingly high hydrogen desorption temperatures, the present compounds exhibit comparatively moderate thermodynamic behavior, indicating their potential for improved hydrogen release characteristics. With negative formation energies, thermal stability at 600 K and absence of imaginary frequency in phonon dispersion analyses, both compounds crystallize in a cubic *Fm*-3*m* symmetry, demonstrating their thermodynamic, thermal and dynamical stability. Mechanical analyses reveal that Mg_2_LiTiH_6_ exhibits ductile character, whereas Mg_2_LiVH_6_ shows slightly brittle traits, though both meet Born stability criteria and offer sufficient rigidity. Rapid hydrogen sorption and desorption are supported by metallic behavior and high carrier mobility, which are confirmed by electronic band structure investigation. High static dielectric constants and substantial absorption in the IR range are optical characteristics that highlight these hydrides' multifunctional potential. The gravimetric hydrogen storage capacities of Mg_2_LiXH_6_, approaching the U.S. DOE 2025 target of 5.5 wt%, together with their suitable desorption temperatures, highlight their suitability for future H_2_ storage devices. The estimated temperatures further suggest that Mg_2_LiTiH_6_ may enable comparatively easier hydrogen release than Mg_2_LiVH_6_, reflecting differences in hydrogen binding strength within the lattice. Importantly, the present results reveal a compositional design principle in which transition metal substitution (Ti *vs.* V) provides an effective route to tune hydrogen binding strength, thermodynamic behavior, and desorption characteristics in Mg_2_Li-based hydrides. This highlights the potential of Mg–Li double perovskite frameworks as a tunable platform for designing hydrogen storage materials with tailored properties through targeted selection of transition metal species. These findings imply that Mg_2_LiXH_6_ hydrides are excellent contenders for upcoming hydrogen storage and energy delivery systems because they provide a promising blend of high hydrogen storage capacity, thermodynamic viability and structural integrity.

## Ethical approval

All authors affirm that this submission is entirely original, has not been published elsewhere, and fully adheres to the established ethical standards for scholarly research and publication.

## Author contributions

All authors affirm their substantial contributions to this research and collectively assume full responsibility for its content, encompassing conceptualization, computational analysis, manuscript preparation, and revision processes.

## Conflicts of interest

The authors declare that there are no financial or personal relationships that could have influenced the outcomes, interpretation, or conclusions presented in this study.

## Data Availability

The data will be provided on request by the corresponding author.
